# A Comprehensive Review on the Interaction Between the Host GTPase Rab11 and Influenza A Virus

**DOI:** 10.3389/fcell.2018.00176

**Published:** 2019-01-09

**Authors:** Maria João Amorim

**Affiliations:** Cell Biology of Viral Infection Lab, Instituto Gulbenkian de Ciência, Oeiras, Portugal

**Keywords:** influenza A virus, viral inclusion, viral assembly, Rab11 GTPase, influenza supramolecular genomic complex

## Abstract

This year marks the 100th anniversary of one of the deadliest pandemic outbreaks, commonly referred as the Spanish Flu, that was caused by influenza A virus (IAV). Since then, IAV has been in governmental agendas worldwide, and a lot of effort has been put into understanding the pathogen’s lifecycle, predict and mitigate the emergence of the strains that provoke yearly epidemics and pandemic events. Despite decades of research and seminal contributions there is still a lot to be investigated. In particular for this review, IAV lifecycle that takes place inside the host cell is not fully understood. Two steps that need clarification include genome transport to budding sites and genome assembly, the latter a complex process challenged by the nature of IAV genome that is divided into eight distinct parts. Assembly of such segmented genome is crucial to form fully infectious viral particles but is also critical for the emergence of viruses with pandemic potential that arise when avian and human IAV strains co-infect a host. The host GTPase Rab11 was separately implicated in both steps, and, interestingly these processes are beginning to emerge as being intimately related. Rab11 was initially proposed to be involved in the budding/release of IAV virions. It was subsequently shown to transport progeny genome, and later proposed to promote assembly of viral genome, but the underlying bridging mechanism the two is far from clear. For simplicity, this Rab11-centric review provides an initial separate account of Rab11 involvement in genome transport and in assembly. IAV genome assembly is a complicated molecular biology process, and therefore earned a dedicated section on how/if the viral genome forms a genomic supramolecular complex. Both topics present intricate challenges, outstanding questions, and unique controversies. At the end of the review, I will explore possible mechanisms intertwining IAV vRNP transport and genome assembly. Importantly, Rab11 has recently emerged as a key factor subverted by evolutionary unrelated viral families (*Paramyxo*, *Bunya*, and *Orthomyxoviruses*, among many others) and bacteria (*Salmonella* and *Shigella*) relevant to human health. This review provides a framework to identify common biological principles among the lifecycles of these pathogens.

## Influenza a Zoonosis and Human Health

Influenza viruses are members of the *Orthomyxoviridae* family. This family was recently updated by the International Committee on Taxonomy of Viruses (ICTV, 2017) and at the moment accommodates seven genera (and nine species): Alpha influenza viruses (IAV), Beta influenza viruses (influenza B virus), Delta influenza viruses (influenza D virus), Gamma influenza viruses (influenza C virus), Isavirus (Salmon isavirus), Quaranjavirus (Johnston Atoll quaranjavirus, Quaranfil quaranjavirus) and Thogotovirus (Dhori thogotovirus and Thogoto thogotovirus) (ICTV, 2017). Influenza C, D, and Thogoto viruses may very rarely cause infections in humans (Lambert et al., 2015; White et al., 2016; Nesmith et al., 2017). Contrarily, incursions of influenza A and B viruses in humans are frequent, as these virus cause yearly epidemics, being relevant pathogens for human health. According to the World Health Organization, these viruses are important contributors of lower respiratory infections and in 2016 occupied the 4th place of cause of death worldwide. Influenza virus A and B genera differ in host range and pathogenicity, and IAVs are the only viruses that pose a significant risk of zoonotic infection, host switch, and the generation of pandemics (Kuiken et al., 2006; Horman et al., 2018; Mostafa et al., 2018), being the focus of this review. Influenza A is a zoonotic virus, widespread throughout highly diverse ecological niches as it infects various animals including humans, pigs, bats, horses, and wild aquatic birds, the latter being the primary reservoir for most IAV (Yoon et al., 2014). The diversity among IAV has led to the establishment of categories based on the viral antigens, i.e., the two main proteins at the surface of the virus: hemagglutinin (H or HA) and neuraminidase (N or NA). There are 18 different hemagglutinin and 11 neuraminidase subtypes (Tong et al., 2013), with 16 HA and 9 NA being found in aquatic birds (Webster et al., 1992) and the remainder in bats. Influenza A in its reservoir infects the intestinal tract, causing mild to asymptomatic disease although with some cost to the animal, including reduced migratory capacity (van Gils et al., 2007). Interspecies transmission is thought to occur mainly from wild to domestic birds (Shortridge, 1992), from where it disseminates to other species (Lam et al., 2013). Many adaptations are required to permit establishment in different hosts which have not been fully understood (Schrauwen and Fouchier, 2014). In hosts other than the reservoir, the disease is more confined to the respiratory tract, although, in cases of highly pathogenic and virulent strains a systemic infection could be established. The high frequency of circulating influenza viruses in diverse animal populations increases the potential for emergence of pandemic virus. These strains have, however, thus far been limited to interspecies transmission from avian and swine viruses to humans (Taubenberger and Kash, 2010; Vijaykrishna et al., 2011).

This year marks the 100th anniversary of one of the deadliest pandemic outbreaks, the 1918 Spanish Flu that was caused by an avian IAV. This virus infected one third of the world population (500 millions), provoking 20–50 million deaths (Johnson and Mueller, 2002). Unusual circumstances underpinned the deadly proportions of the outbreak, including the first world war that devastated populations, causing poverty, malnutrition, poor sanitation, overcrowding and massive troop movements, as well as excessive contact with animals (Wever and van Bergen, 2014). In addition, lack of suitable therapeutic and health care means, and absence of pre-existing immunity to the zoonotic virus, combined with an unusual pathogenicity of the viral strain contributed to excess mortality and spread of the disease. Since then, three additional influenza pandemics of severe consequences have been reported: the 1957 H2N2 ‘Asian Flu’ pandemic (0.7 million–1.5 million deaths) (Viboud et al., 2016), the 1968 H3N2 “Hong Kong Flu” (1 million deaths) (Mathews et al., 2009), and most recently, the 2009 novel H1N1 virus causative of “Swine flu” (151,700–575,500 deaths) (Dawood et al., 2012). Of note, pandemic outbreaks have been and are still impossible to predict. Key aspects favoring the incursions of influenza in humans include its, already mentioned, widespread distribution, and viral evolution. Establishment of a virus in a specific host requires adaptation to that host, rendering viral sub-types restricted to particular animal species (Taubenberger and Kash, 2010; Schrauwen and Fouchier, 2014). IAV evolution is fast with different contributor factors (Lowen, 2018). The viral polymerase has low replicative fidelity that results in an average of 2–3 mutations per replicated genome, and therefore the viral progeny consists of genetically diverse population (termed quasispecies) (Pauly et al., 2017), containing many defective virions. The increased risk of producing defective or semi-infectious progeny viruses associated with the viral polymerase is largely compensated by the emergence of escape mutants that bypass neutralizing antibodies, host immunity and antiviral drugs. In addition, the number of quasispecies is increased by the segmented genome. The segmentation of the viral genome permits mixing of different parental strains in co-infections, a process known as reassortment. Reassortment originates chimeric viral genomes, where one (or more) entire segments are swapped when two different viruses co-infect the same cell. The genetic mixing in infected cells can lead to the emergence of viruses with pandemic potential, especially when genome recombination occurs between viruses adapted to different species. The advantages of having an eight-partite genome are evident for viral evolution, although the segmented genome increases the complexity of the assembly of fully infectious virions. The way this genome assembles is very relevant to human health and, in part, the topic of this review. The subject is controversial and has many unresolved questions, despite decades of dedicated research. Here, I will explore the knowns, unknowns and currently under debate views, and for this I will briefly introduce some aspects of the structure of the virion, lifecycle and mechanisms of viral genome assembly.

## Influenza a Virion Structure

**Figure 1:**
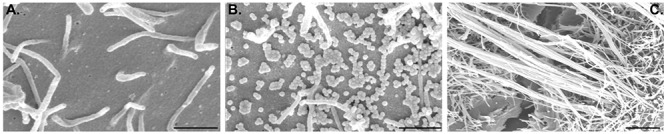
Apical surface of A549 lung epithelial cells healthy **(A)** or infected with influenza A/Puerto Rico/8/34 **(B)** or a reassortant of this virus with segment 7 A/Udorn/301/72 **(C)**. This figure aims to show the different morphologies of influenza A virion budding from cells [spherical **(B)** and filamentous **(C)**]. Scale bar is 1 μm.

Lab adapted influenza A viral strains are typically small and spherical, with diameters of ∼100 nm (Figures 1A,B). Clinical isolates, however, when initially analyzed in the lab display a range of morphologies with the prevalent form being spherical particles, but presenting many other morphologies described as filaments [for comprehensive review (Dadonaite et al., 2016)] and bacilliform (Vijayakrishnan et al., 2013) (Figure 1C). Spherical and bacilliform virions contain, in 80% of the cases, complete genomes (Nakatsu et al., 2016). Filaments can be very long (over 10 μm) and homogeneous in width (100 nm), or heterogeneous with a bulbous head with a diameter greater than 200 nm known as Archetti body (Archetti, 1955), and variable lengths. Interestingly, many of these irregular shaped and long budding virions lack viral genomes (Vijayakrishnan et al., 2013).

**Figure 2:**
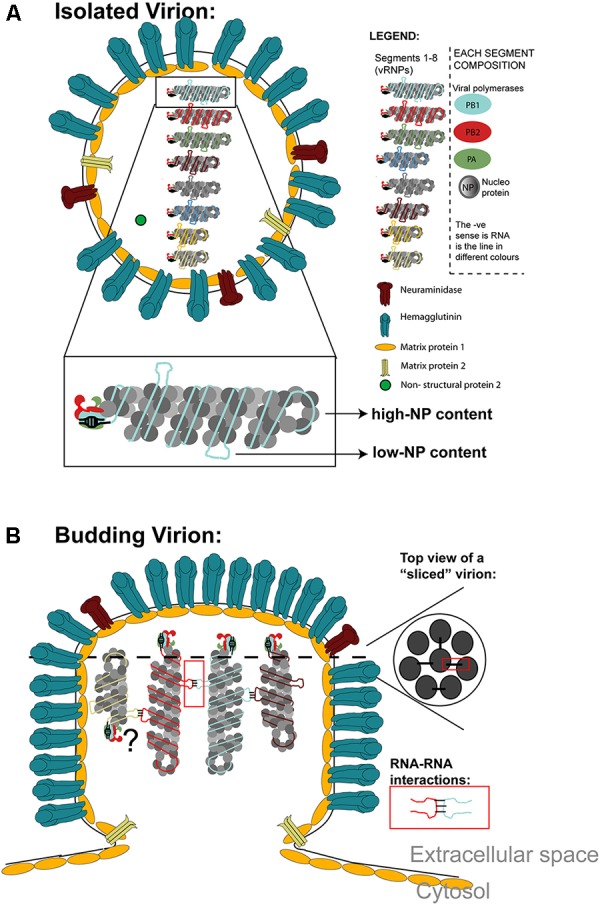
**(A)** Schematics of a virion containing the plasma-membrane derived envelope containing the three transmembrane proteins: M2, HA, and NA. Beneath the plasma membrane, the M1 protein forms a tight layer and the virion core contains the 8 vRNP segments that constitute its genome and very small amounts of NS2. vRNPs are magnified to illustrate their structure with the heterotrimeric RNA dependent RNA polymerase constituted by PB1, PB2, and PA docking at the panhandle structure, and NP coating RNA forming a double-helical hairpin structure, with a loop at one end. There are regions of high and low NP content. Low NP content regions are available to establish RNA–RNA interactions among the different vRNP types. Note that cellular proteins are omitted from this model. **(B)** Schematics of a budding virion is shown. The vRNP distribution inside virions is shown on lateral- or top-view sliced sections, the latter showing the vRNP arrangement “7+1.” vRNPs bind to the top of the virion and have been reported to adopt parallel and anti-parallel orientations, the latter being still controversial (hence the question mark). vRNPs should establish RNA-RNA interactions within different vRNPs (displayed in the red square).

The virion illustrated in Figure 2A contains a host derived plasma membrane that harbors three viral transmembrane proteins unevenly distributed and present in non-stoichiometric amounts. HA is involved in viral entry, being the most abundant of the three and is a glycoprotein that forms trimers (Copeland et al., 1986). NA cleaves sialic acids being involved in viral exit. It is also a glycoprotein, the second in abundancy, and forms tetramers that cluster (Harris et al., 2006; Calder et al., 2010; Wasilewski et al., 2012; Hutchinson et al., 2014; Chlanda et al., 2015). The proton channel M2 is the least abundant viral transmembrane protein (Hutchinson et al., 2014), playing a crucial role in the release of viral material from endosomes (Kemler et al., 1994). Beneath the lipid membrane is a layer of the viral protein M1 that plays a determinant role in virion morphology (Roberts et al., 1998). M1 oligomerizes to form a helical matrix with a range of curvatures (Calder et al., 2010). The virion core accommodates a segmented viral genome composed of eight segments, and will be described in detail below. The genome displays an arrangement with seven external segments surrounding a central RNA piece (7+1 configuration) in Figure 2B (top view) (Oxford and Hockley, 1987; Noda et al., 2006). In most cases, the eight segments are found hanging from the tip of the virion and it is still controversial if they can adopt anti-parallel orientations (Sugita et al., 2013). IA virions incorporate selected host proteins, some shared by all influenza strains, and others strain and host specific (Hutchinson et al., 2014).

### Outstanding Questions Regarding Virion Structure

#### Host Protein Incorporation in Virions Is Not Well Understood

It is well established that IA virions contain host proteins (Shaw et al., 2008; Hutchinson et al., 2014), some of which could provide advantage to the virion by stabilizing the particle or conferring resistance to host immunity, as is the case of distinct host complement inhibitor molecules in many virions (Favoreel et al., 2003). Studies indicate that there is a selection of host proteins incorporated in virions, although the identity of these proteins and their roles in infection is ill defined. In addition, the mechanisms responsible for the selection of virion-included proteins are far from clear.

#### The Role of the Different Shaped Influenza Virions Is Not Established

Despite the efforts done to explore the composition and formation of the distinct influenza A virion morphologies [for review (Dadonaite et al., 2016)], it is unclear if the different shaped-influenza virions are ornamental by-products of infection or have functional roles. An answer to this question could, in principle, be applied to all members of the *Orthomyxoviridae* family as well as many other respiratory viruses, all shown to produce filaments.

#### How Do Host and Viral Proteins Cooperate for the Budding and Shaping of Virions?

Virions are formed at the plasma membrane using host and viral constituents [for reviews check (Rossman and Lamb, 2011; Hutchinson and Fodor, 2013)]. Many proteins, lipids (including cholesterol) and the fully assembled genome (Amorim et al., 2013; Gavazzi et al., 2013b) have been shown to be essential or contribute to accelerate or facilitate the budding process, but how they cooperate to propel both budding and virion shape remains far from elucidated.

## Structure of Viral Ribonucleoprotein (vRNP) Complexes and IAV Genome

The genome is divided into eight segments of single-stranded, negative-sense RNA that vary in length from ∼0.9 to ∼2.3 kb having a total size of ∼13.5 kb. Each segment (illustrated in Figure 2A) is encapsidated as individual rod-like vRNPs (Pons et al., 1969) of 10–15 nm in diameter and 30–120 nm in length (Compans et al., 1972; Noda et al., 2006). All vRNPs are composed of one copy of the heterotrimeric RNA-dependent-RNA-polymerase (RdRp) formed by PB1 (catalytic unit), PB2 and PA (priming transcription units) (Horisberger, 1980; Honda et al., 1990; Guilligay et al., 2008; Yuan et al., 2009), viral nucleoprotein (NP) and viral RNA. The 5′ and 3′ ends of the RNA are composed of conserved RNA sequences of 13 and 12 nucleotides that display partial inverted complementarity and base-pair in an antiparallel manner to form an approximately 15-base pair-long panhandle (Detjen et al., 1987), where the RdRp assembles (Murti et al., 1988). As a result, a double-helical hairpin structure is formed, with a loop at one end folding back on itself (Pons et al., 1969; Hsu et al., 1987). The double-helix contains major and minor grooves caused by the oligomerization of NP that forms a trail of positive residues enabling binding of RNA to the outside of the complex (Ye et al., 2006, 2012; Arranz et al., 2012; Moeller et al., 2012), but rendering vRNA susceptible to RNAse digestion (Yamanaka et al., 1990). Studies showed that 2 molecules of NP interact with PB1 and PB2 (Biswas et al., 1998; Martin-Benito et al., 2001; Arranz et al., 2012; Moeller et al., 2012). The periodicity of NP on RNA is ∼21 nucleotides (Williams et al., 2018). Recent studies show that the ssRNA is coiled around the NP monomers in an unevenly fashion, with some regions tightly bound to NP and others with NP dynamically turning over and therefore exposed (Moeller et al., 2012; Gallagher et al., 2017; Lee et al., 2017; Williams et al., 2018). High-NP content is prevalent on G-rich and U-poor RNA regions, indicating some sort of sequence bias for NO binding as analyzed by HITS-CLIP (Lee et al., 2017) and low-NP content is found in predicted RNA secondary structures by PAR-CLIP (Williams et al., 2018). These regions were proposed to be functionally linked to efficient vRNP packaging in virions (Lee et al., 2017; Williams et al., 2018) with synonymous mutations attenuating influenza replication (Williams et al., 2018).

vRNPs are individual replication units of a specific segment, being able to transcribe and replicate the encoded material under appropriate conditions (Pleschka et al., 1996; Mullin et al., 2004). The way vRNPs are incorporated or packaged in a virion, assembled into a genome, and transmitted/received by neighboring cells/individuals is a key parameter in viral perpetuation. In the case of influenza, as mentioned, this is particularly challenging as the viral genome is segmented, which imposes additional layers of complexity to the assembly of the viral genome. Two models were proposed for the inclusion or packaging of individual vRNPs into budding virions: the random and the selective models. The first model implies that a complete genome is formed by chance without any mechanism to distinguish between different segments. The selective model accommodates a mechanism whereby each virion packages one copy of each unique vRNP (Kingsbury, 1970; Compans et al., 1972). To understand the current situation where the data overwhelmingly favors the selective packaging model, readers are remitted to this comprehensive review (Hutchinson et al., 2010). Briefly, studies demonstrating competition between full-length segments and defective interference particles (segments that have internal deletions) (Davis et al., 1980; Jennings et al., 1983; Duhaut and McCauley, 1996; Duhaut and Dimmock, 1998), and genetic and biochemical analyses revealing that the influenza A genome is haploid, started paving the way favoring the selective model (Hatada et al., 1989; Fujii et al., 2003). Other important contributing studying used reverse genetics, mapped regions responsible for the packaging of specific segments (Duhaut and Dimmock, 2000; Muramoto et al., 2006; Gog et al., 2007; Hutchinson et al., 2008, 2009; Essere et al., 2013; Gavazzi et al., 2013b). Final evidence in favor of the selective model was the visual inspection of virions by electron microscopy/tomography revealing eight segments inside the virion (Noda et al., 2006; Fournier et al., 2012b; Noda et al., 2012; Sugita et al., 2013) with no indication of incorporation of excess number of segments (Harris et al., 2006; Yamaguchi et al., 2008). Seminal studies have captured a 7+1 configuration of vRNPs inside budding virions at the plasma membrane (see Figure 2B). The differences in vRNP length (as a consequence of different number of nucleotides) permitted ranking vRNPs by length into three categories by 3D-tomography: Segments 1 – 3 as long; segments 4 – 6 as medium and segments 7 and 8 as small. Assessment of segment length indicated that the 80% of virions incorporated one complete genome set (Nakatsu et al., 2016). Despite the strong evidence for a stringent, selective model, these data do not constitute proof of either a specific layout of vRNPs in virions or of the establishment of inter-segment RNA interactions. Recently, however, interactions between RNPs have been observed suggesting that the genome actually forms a supramolecular complex (Fournier et al., 2012a,b; Noda and Kawaoka, 2012), held together by base pairing of packaging signals on RNA (Fournier et al., 2012b). Until the present date, it is accepted that RNA–RNA (Essere et al., 2013; Gavazzi et al., 2013a,b), but not protein-RNA interactions, guide genome packaging. Mechanistically, it was proposed that secondary structures on vRNPs (Ferhadian et al., 2018), and specially in conserved regions of low-NP content could allow the establishment of non-covalent inter-segment interactions likely by hybridization (Lee et al., 2017; Williams et al., 2018). After released from the cells, vRNPs in isolated virions studied by cryo-tomography were shown to maintain the double-helical structure and remain attached to one end of filamentous particles (Harris et al., 2006; Calder et al., 2010; Wasilewski et al., 2012; Vijayakrishnan et al., 2013). In terms of morphology, RNP filaments varied in length, curvature and handedness of the helix, not being uniformly rigid structures (Arranz et al., 2012; Moeller et al., 2012; Gallagher et al., 2017) on account of NP-NP interactions (Gallagher et al., 2017).

### Unresolved Questions Regarding vRNP Structure in a Virion

#### Do All Virions Contain a Copy of Each vRNPs?

This is a controversial aspect of IAV infection. Many reports have shown that most infected cells fail to express all viral proteins (Brooke et al., 2013; Brooke et al., 2014; Heldt et al., 2015). This data is consistent with two scenarios: On the one hand, a faulty genome assembly whereby virions fail to package a complete genome by means of lacking segments or repeating the packaging of the same genomic piece; on the other hand, virions could incorporate segment(s) with null mutation(s) unable to express the encoded protein(s) by a variety of different processes that include (among others) non-sense mutations or loss of ability to interact with host machinery for being delivered to specific sites or to enable transcription/replication. Compelling scientific evidence is required to clarify this important aspect of viral transmission regulation.

#### Does IAV Genome Form a Supramolecular Complex?

Seminal work established the requirement of *cis*-acting and intersegment RNA–RNA interactions in the formation of the eight-vRNP viral genome [for reviews check (Ferhadian et al., 2018)]. In addition, cryo-tomography of budding virions captured images of inter-connected segments (Fournier et al., 2012a,b; Noda and Kawaoka, 2012; Gavazzi et al., 2013a) and recent preprint data from Fodor Lab using PAR-CLIP (Dadonaite et al., 2017) found biochemical evidence in support of existing inter-segment ligations in isolated virions. Together, these studies push forward the notion that IAV genome actually forms a complex with strong interactions among segments. The next step would be to predict genome arrangements in virions based on viral sequences, cell types and host species and to identify where the assembly of IAV genome takes place.

## The Circuit of vRNPs in IAV Infected Cells

Influenza A virus initiates infection upon binding of hemagglutinin to sialic acid on transmembrane proteins on the host cell surface, entering via receptor-mediated endocytosis. As pH drops during endosome maturation, the membranes of the virus and of the endosome fuse and the proton pump activity of the viral protein M2 allows the release of vRNPs to the cytosol. vRNPs are subsequently transported to the nucleus where transcription and replication of influenza RNA occurs. As already mentioned, each segment constitutes an independent transcription-replication unit equipped with its own polymerase (Wise et al., 2009; Dou et al., 2018), with the same vRNA template being transcribed into capped and polyadenylated viral mRNA or copied as a complementary RNA (cRNA). cRNA will then serve as template to produce multiple copies of vRNA as reviewed in (Fodor, 2013; Hutchinson and Fodor, 2013; Te Velthuis and Fodor, 2016; Pflug et al., 2017). vRNP nuclear export requires a daisy chain reaction using the viral proteins M1 and NS2 and the cellular protein CRM1 (Martin and Helenius, 1991; O’Neill et al., 1998; Neumann et al., 2000; Elton et al., 2001; Watanabe et al., 2008, 2014). Upon exiting the nucleus, vRNPs accumulate around the microtubule organizing centre (MTOC) (Momose et al., 2007; Bruce et al., 2010; Jo et al., 2010; Amorim et al., 2011; Eisfeld et al., 2011a; Momose et al., 2011; Avilov et al., 2012b). Subsequently, vRNPs are transported to the plasma membrane by unclear mechanisms. Together with host and the other viral major structural proteins (M1, M2, HA, and NA), vRNPs are packaged into progeny virions, which bud and are released from the cell [reviewed recently in (Pohl et al., 2016; Dou et al., 2018)].

## Rab11 Cycle in Healthy Cells

Transport of the vRNPs to the cell surface, assembly of influenza A genome into a supramolecular complex, virion budding and release from the cell have been the subject of many recent seminal manuscripts, many of which involving the host GTPase Rab11 [as reviewed in (Hutchinson and Fodor, 2013; Gerber et al., 2014; Eisfeld et al., 2015; Giese et al., 2016; Lakdawala et al., 2016; Pohl et al., 2016; Dou et al., 2018)]. Interestingly, Rab11 is emerging as a key factor subverted by evolutionary unrelated viral families (*Paramyxo*, *Bunya*, and *Orthomyxoviruses*, among many others) and bacteria (*Salmonella* and *Shigella*) of relevance to human health. The interactions and role of Rab11 in bacterial and viral infections are explored in these reviews (Guichard et al., 2014; Vale-Costa and Amorim, 2016). Importantly, Rab11 function in several infections might hold common biological principles that could be explored therapeutically. It is therefore important to obtain a roadmap of Rab11 behavior and interactions for each infection. This review deals with the current knowledge, challenges and controversies of the interaction between Rab11 with IAV.

Rab11 is a member of a large family of GTPases called Ras-related in brain (Rab) with 44 subfamilies encoded in the human genome (Diekmann et al., 2011). The activity of every Rab depends on GDP/GTP association: the GTP-bound active form and the GDP-bound inactive form. A guanine nucleotide exchange factor (GEF) catalyzes GTP-binding and causes a conformational change. The GTP-bound conformation allows recruitment of molecular motors, tethers and SNAREs to drive, dock and fuse, respectively, vesicles to their cognate membranes (Stenmark, 2009). It is the association of lipids and proteins in the vesicles with the ones in organelles that ensures the high fidelity of the vesicular transport, and delivery of material where and when is needed [reviewed in (Vale-Costa and Amorim, 2016)]. During transport, tethering and fusion, Rabs are converted back to the GDP-bound form through hydrolysis of GTP, stimulated by a GTPase-activating protein (GAP) [with one inorganic phosphate (P_i_) being released]. In the GDP-bound form, Rabs can be recycled back to specific membranes via the action of many different enzymes (Diekmann et al., 2011; Hutagalung and Novick, 2011).

Rab11 subfamily is composed of Rab11 a, b, and c, the latter also called Rab25. Rab11a and b, that share 89% homology, have been implicated in IAV lifecycle, although Rab11a has been studied in more detail and for this reason all subsequent mentions of Rab11 refer to the Rab11a isoform. In uninfected cells, Rab11 is the master regulator of the endocytic recycling compartment (ERC), one of the systems the cell uses for delivering endocytosed material, as well as, specific cargo from the TGN, to the cell surface (Grant and Donaldson, 2009; Vale-Costa and Amorim, 2016). It has been shown that in healthy cells, the ERC derives from early endosomes by the action of KIF13A (Delevoye et al., 2014) and actin (Ripoll et al., 2016) and whether there is a pool of ERC-vesicles derived from the TGN formed in a distinct manner is not known.

In healthy conditions, Rab11-GTP recruits a series of molecular motors using molecular adaptors called Rab11-family interacting proteins (FIPs) (Horgan and McCaffrey, 2009) to drag vesicles on cytoskeletal tracks, but also to attach vesicles to transition zones between microtubules and actin. Rab11 containing vesicles tether to the plasma membrane using SEC15, a member of the exocyst, or Munc13-4. Finally, the fusion step at the plasma membrane involves SNAP25 (a SNARE), and/or SYN4 and/or VAMP8 (Wallace et al., 2002; Aikawa et al., 2006; Sato et al., 2008; Collins et al., 2012; Schafer et al., 2014; Marshall et al., 2015; Johnson et al., 2016).

## Functional Relevance of Rab11 During IAV Infection

Many Rabs, including Rab5, Rab7, and Rab9 were shown to be important for IAV infection. Assessment of their role was straightforward, as their depletion would affect viral entrance (Lakadamyali et al., 2003). Conversely, the function of Rab11 in IAV infection has been difficult to demonstrate and is still under debate. Rab11 was shown to affect viral production, especially when Rab11a and Rab11b were knocked down simultaneously, being stipulated that these factors are required for efficient viral replication (Bruce et al., 2009). When assessing the step in which Rab11 was required, studies have consistently found defects only in the late stages of infection. It was proposed that Rab11 transported vRNPs and promoted assembly of the genome and that the processes were intimately related. However, the molecular mechanisms bridging the two steps in the viral lifecycle are controversial. For simplicity, I will separately introduce involvement of Rab11 in vRNP transport (see section “Rab11 and vRNP Transport”) and IAV genome assembly (see section “Briding vRNP Transport With Genome Assembly”) and will finalize exploring the bridging mechanisms that are currently under evaluation (see “Briding vRNP Transport With Genome Assembly”).

### Rab11 and vRNP Transport

As stated above, the function of Rab11 in healthy cells is to transport endocytosed and specific sets of TGN derived vesicles to the cell surface. Mechanistically, Rab11 in vesicles attracts several molecular motors and moves on microtubules and actin [for review (Vale-Costa and Amorim, 2016)]. In IAV infection, if Rab11 delivers vRNPs to the cell surface is controversial in multiple ways.

First, it is unclear the extent of microtubule contribution to movement of vRNP-laden Rab11 vesicles during IAV infection. In 2011, several studies were published using live cell imaging systems of cells infected with fluorescent tagged viruses, overexpressing GFP-NP, split-GFP or injected with antibodies targeting vRNPs to track the dynamics and behavior of progeny IAV genomes. It was found that vRNPs could diffuse or use actin and microtubules, the latter promoting fast movements, in synchrony with Rab11-GTP. In addition, in the absence of Rab11, vRNPs exhibited different kinetics (Momose et al., 2007; Jo et al., 2010; Amorim et al., 2011; Eisfeld et al., 2011b; Momose et al., 2011; Avilov et al., 2012a,b). This led to a model whereby vRNPs exited the nucleus to be loaded on Rab11 vesicles and transported to the plasma membrane using, at least in part, microtubules for fast transport. Supportive data included the identification of: (1) modifiers of microtubules, as for example YB-1, that positively impacted both in vRNP loading on Rab11 and in Rab11 association with microtubules (Kawaguchi et al., 2015); and of (2) HRB, a protein with ARF GAP activity, able to regulate vRNP trafficking from the perinuclear region to the plasma membrane (Eisfeld et al., 2011b). Discrepant results included the modest reduction in viral titers when microtubules were depolymerized, and decrease in Rab11 binding to FIP adaptors upon infection, suggesting that association between Rab11 vesicles and microtubules was compromised (Amorim et al., 2011; Momose et al., 2011; Vale-Costa et al., 2016). In an attempt to reconcile the function of Rab11 in healthy and infected cells, it was suggested that redundant mechanisms cooperated to transport vRNPs to the surface. In agreement, a recent study showed that vRNP subcellular location could be uncoupled from Rab11 in the presence of nocodazole (Nturibi et al., 2017). Whether both types of transport (Rab11-dependent vs. Rab11-independent) co-exist remains to be demonstrated.

Second, a large body of evidence suggests that the Rab11 cycle described in section 5 is modified during IAV infection. In fact, Rab11 distribution (Amorim et al., 2011; Eisfeld et al., 2011a; Momose et al., 2011; Avilov et al., 2012a), ultrastructural environment (Amorim et al., 2011; Kawaguchi et al., 2015; de Castro Martin et al., 2017), function (Kawaguchi et al., 2015; Vale-Costa et al., 2016) and binding partners (Vale-Costa et al., 2016) were shown to be altered with IAV infection. Multiple techniques for visualizing proteins in cells either in live systems or frozen snapshots showed that, in uninfected cells, Rab11 subcellular distribution is cytosolic, dispersed in small dots. However, in infection, and dependent on the presence of progeny vRNPs in the cytosol (Vale-Costa et al., 2016), the distribution of Rab11 is altered, initially concentrating around the perinuclear region (around the MTOC mostly) to then originate enlarged puncta dispersed through the cytosol where vRNPs also co-localize (Amorim et al., 2011; Eisfeld et al., 2011a; Momose et al., 2011; Avilov et al., 2012b). Ultrastructural inspection of sites positive for Rab11 and vRNPs, using correlative light an electron microscopy, showed that enlarged puncta were formed by clustered vesicles of heterogeneous sizes and from where coiled-coil structures protruded. These structures were similar in size (Vale-Costa et al., 2016), morphology and shape to vRNPs in budding virions, (Noda et al., 2006; Fournier et al., 2012b; Noda and Kawaoka, 2012; Sugita et al., 2013). In a more recent manuscript, these vesicles were renamed irregular coated vesicles (de Castro Martin et al., 2017). Importantly, the concentration of viral (and cellular) material in a cellular location, regardless of harboring viral replication, is denominated viral inclusion, and this nomenclature will be interchangeably applied in the manuscript together with enlarged puncta/clustered vesicles. Functionally, several independent reports showed that the composition of Rab11-vesicles of infected cells was different to that in healthy cells, with many vesicles devoid of transferrin receptor (de Castro Martin et al., 2017), enriched in cholesterol (Kawaguchi et al., 2015), or accumulating transferrin receptor preferentially at the pericentriolar ERC (Kawaguchi et al., 2015; Vale-Costa and Amorim, 2016). Together, these results indicate that infection impairs ERC vesicular movement and function, and that Rab11 works in a distinct manner.

Third, it is unclear if Rab11 is the final carrier of vRNPs to the cell surface. The lack of Rab11 at the surface of IAV infected cells, or inside virions led to the hypothesis that Rab11 was a way station and that vRNPs were handed-over to a different host protein for final delivery to the cell surface (Eisfeld et al., 2015). However, a recent manuscript proposed an alternative model in which once progeny vRNPs reached the cytosol they first bind to a modified endoplasmic reticulum (ER) from where Rab-11 coated vesicles loaded with vRNPs would subsequently be released and directed to the plasma membrane (de Castro Martin et al., 2017). In this latter scenario, Rab11-vesicles would be the final transporters of vRNPs to the plasma membrane, conflicting with the lack of Rab11 in virions and at the plasma membrane, but consistent with a modest role for microtubules during infection. Supporting data for an involvement of the ER includes a preprint manuscript showing that viral inclusions form in the vicinity of ER exit sites (ERES) in a way that is dependent on continuous cycles between the ER and the Golgi (Alenquer et al., 2018).

Future research is needed to clarify the mechanisms underlying vRNP transport to the plasma membrane.

### Rab11 and IAV Genome Assembly

In light of recent reports demonstrating the formation of an IAV supramolecular genomic complex (Fournier et al., 2012b; Noda et al., 2012; Dadonaite et al., 2017), it is crucial to understand the cellular locations and the mechanisms whereby the genome complex is formed.

**Figure 3:**
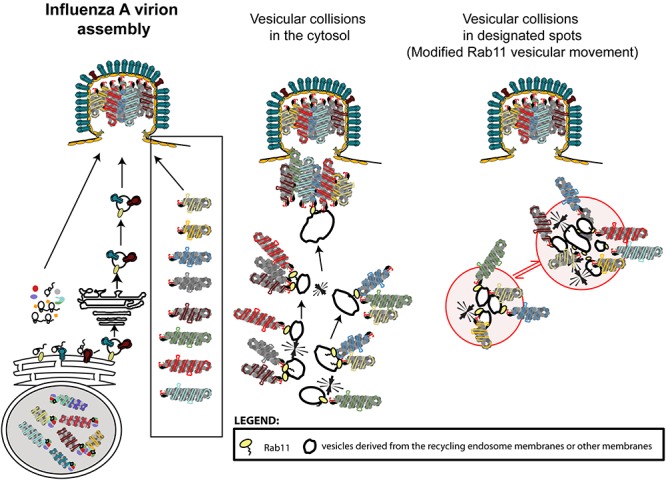
Influenza A virion assembly schematics. Virion components must reach the apical side of the plasma membrane. Proteins including M1 are translated in the cytosol and bind the transmembrane proteins at the cell surface. Transmembrane proteins are translated in the rough ER and reach the lipid membrane using the classic secretory pathway. vRNPs are found inside virions displaying a 7+1 arrangement and forming a complex. Current models suggest that the genomic complex is formed before reaching the cell surface by the establishment of RNA–RNA interactions promoted as vesicles transporting vRNPs collide throughout the cytosol (dispersed vesicular collision model) or in designated locations (compartmentalized model, with the compartment shown as red circles on the left). Note that formation of designated locations would require alterations in vesicular transport and development of structures where material exchange between the cytosol and viral inclusions could occur. The models presented are an over-simplification and, in both cases, additional steps could be included. Host proteins, with the exception of Rab11 have been omitted from the model.

The currently accepted model, widely disseminated in several reviews (Hutchinson and Fodor, 2013; Gerber et al., 2014; Eisfeld et al., 2015; Giese et al., 2016; Lakdawala et al., 2016; Pohl et al., 2016; Dou et al., 2018), couples genome transport to genome assembly. This dispersed (vesicular collision) model predicts that whilst on-route to the plasma membrane, cytosolic Rab11-vesicles carrying vRNPs collide transiently, allowing the establishment of RNA-RNA interactions (Figures 3, 4.1.1). Several microscopy-based approaches support this model, including the well-documented formation of viral inclusions that expand and accommodate a higher number of vRNP segments during the course if infection (Amorim et al., 2011; Avilov et al., 2012a; Chou et al., 2013; Lakdawala et al., 2014). However, the involvement of ER in vRNP transport and the accumulating evidence on viral-induced alterations to the Rab11 cycle justifies considering alternative mechanisms (Kawaguchi et al., 2015; Vale-Costa et al., 2016; de Castro Martin et al., 2017). A possibility is that vesicular collisions occur between the site of budding of vRNP-Rab11 vesicles from the ER and the plasma membrane (Figure 4.1.2). Another possible mechanism considers that vRNP binding to Rab11 vesicles impairs/re-routes Rab11 (and vRNPs) to specific cellular sites. The concentration of material in such restricted sites could serve to compartmentalize the different vRNP segments, separating them from the rest of the cytosol thus creating designated sites for genome assembly – the compartmentalized model (Figures 3, 4.2.1,4.2.2). The modification/re-routing data of Rab11 cycle during infection is in agreement with the compartmentalized model. Additionally, viral inclusions were shown to be established in cells expressing one single vRNP indicating that the formation of these structures precedes (and is not contingent on) the establishment of RNA–RNA interactions. Furthermore, viral inclusions were shown to have liquid properties segregating from the cytosol without a delimiting membrane, being able to exchange material dynamically, and deform easily as well as to adapt fast to changes in the cellular environment (Alenquer et al., 2018). Therefore, given the liquid properties of viral inclusions and although it is possible that vesicles in these structures collide to establish vRNP intersegment interactions, other unidentified processes could also take place.

**Figure 4:**
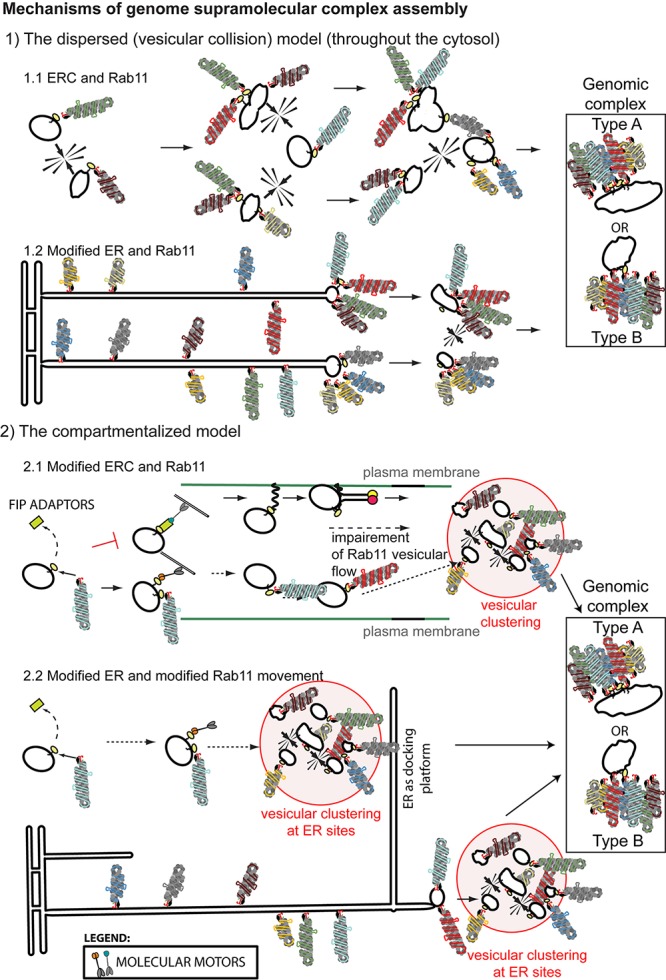
Bridging influenza A vRNP transport with genome assembly. **(1)** The dispersed (vesicular collision) model predicts that genomic complex formation could occur coupled to vRNP transport on Rab11-containing-vesicles. Establishment of inter-segment interactions would take place sequentially during transitory vesicular collision events leading to formation of sub-bundles until the genome complex is completed (with eight different segments). Vesicles could fuse (leading the genomic complex type A) or vRNPs could be transferred from one vesicle to the next upon vesicular “kissing” events (genomic complex type B). Note that type A and B differs in the number of molecules of Rab11 (with type A having at least 8) and the size of the vesicles (type A having enlarged structures). Several mechanisms are proposed based on the literature: **(1.1)** Collision of ERC vesicles containing Rab11 and vRNPs could take place in the entire cytosol. **(1.2)** Collision of vesicles containing vRNPs and Rab11 but derived from a modified ER would take place close to the plasma membrane. **(2)** The compartmentalized model predicts that viral infection would induce the re-routing or impairment of Rab11 pathway and lead to compartmentalization of the different vRNPs inside viral inclusions as a mechanism to facilitate viral assembly **(2.1)** in multiple docking platforms or **(2.2)** at precise cellular spots defined by the ER. Viral inclusions could be formed if vRNPs are transported: (a) on ERC vesicles to the ER or (b) attached to the ER and subsequently released attached to Rab11 vesicles routed to the plasma membrane.

### Briding vRNP Transport With Genome Assembly

The two models for IAV genome assembly (dispersed versus compartmentalized) are coupled with IAV genome transport and involve Rab11, but use very different molecular mechanisms. As mentioned, the dispersed model is supported by formation of viral inclusions containing increased number of vRNP segments as the distance to the plasma membrane decreases (Chou et al., 2013; Lakdawala et al., 2014). However, it conflicts with the lack of Rab11 at the plasma membrane (Eisfeld et al., 2011a, 2015), and in virions (Hutchinson et al., 2014), as well as with the reduction in Rab11-mediated vesicular movement and induction of vesicular clustering (Kawaguchi et al., 2015; Vale-Costa et al., 2016). Discrepancies with lack of Rab11 in virions could be conciliated by considering that one Rab11 molecule would be required per virion. This would be feasible if the establishment of RNA-RNA interactions was stronger than Rab11 binding, with Rab11-vesicular “kissing” originating vRNP transfer from one vesicle to another, suggesting that not many vesicles (or Rab11) were required at the cell surface (Figure 4, genomic complex type B). The alternative would imply fusion of colliding vesicles, and formation of enlarged vesicles containing several Rab11 molecules and vRNPs (Figure 4, genomic complex type A). Vesicular clustering is difficult to accommodate in this model except if considering that unproductive cross-linking among several colliding vesicles could originate these structures. However, the clustered vesicles identified by electron microscopy match the viral inclusions observed by fluorescent microscopy and these are the basis supporting the dispersed vesicular collision model.

The compartmentalized model predicts that formation of viral inclusions results from impairment, modification or re-routing of Rab11 and consequently of the vesicles Rab11 attaches to. Data supporting this model has been listed and explained in Sections “Rab11 and vRNP Transport” and “Rab11 and IAV Genome Assembly” and is illustrated in Figure 4.2.1. In addition, viral inclusions were shown to display liquid properties, forming membraneless organelles, whose assembly might be spatially regulated. In fact, vRNP/Rab11 crowding occurred in vicinity of ERES and was shown to be dependent on continuous vesicular cycling between ER and Golgi (Figure 4.2.2) (Alenquer et al., 2018). The mechanisms mediating viral inclusion formation or/and Rab11 re-routing to the ER remain to be investigated.

### Outstanding Questions Regarding Rab11 Function in IAV Infection

#### Which Factors Regulate the Formation and Properties of Viral Inclusions?

Influenza A virus inclusions have been detected in a variety of cell types, including fully differentiated human primary bronchial cells and macrophages, which argue for some sort of functionality in infection. However, presently, all assigned roles for viral inclusions have not been formally proved. Aspects that could shed light into the functional relevance of viral inclusions include understanding their formation and properties.

#### What Is the Origin of the Different Membranes That Form the Viral Inclusions?

The nature of the clustered vesicles is still unclear and controversial. Two hypotheses apply: Rab11 binds to membranes from the ERC or Rab11 is re-routed to other organelles including the ER.

#### Is Rab11 the Final Transporter of vRNPs to the Plasma Membrane?

This open question is of utmost importance as it might hold the key to reveal the mechanism of IAV genome assembly.

## Concluding Remarks

In recent years, exciting reports have contributed to advances in understanding IAV genome assembly. It has become accepted that influenza A genome forms a supramolecular complex and basic principles are expected to apply to engineer the 7+1 arrangement vRNPs adopt inside a virion. Being able to predict these principles is important to determine next years’ epidemics strain, as well as foresee viruses with pandemic potential. These studies have traditionally been done by studying vRNP structure, vRNP inter-segment biochemical interactions and assessing geographical locations of specific vRNP types in budding or isolated virions of many different IAV strains and in distinct hosts. However, knowledge about IAV genome assembly also requires fully tackling the molecular mechanisms underlying its formation inside the host cell. Several reports have suggested a model coupling IAV genome assembly with vRNP transport involving the host protein Rab11, whereby “kissing” between vesicles resulted in the establishment of inter-segment interactions. However, this model conflicts with several experimental results and requires more investigation. In particular, a bulk of evidence suggests that Rab11 is modified by infection but the outcome is ill-defined. Recent advances on cellular compartmentalization are revolutionizing the way we understand how spatial organization promotes biological reactions in time and in a densely populated cytosolic environment. This emerging topic might permit explaining sophisticated phenomena such as assembly of segmented viral genomes. More insights will arise from developing approaches to overcome some of the technical limitations we current have including tracking individual vRNPs, or several organelles, or resolving assembled genomes inside the living cell. Finally, innovative models to study viral infection, in a way that takes into account cell polarization without compromising the resolving power inside individual living cells, will also be crucial to understand how proteins that promote trafficking and establish cell polarization are involved in viral assembly.

## Author Contributions

MJA wrote the manuscript.

## Conflict of Interest Statement

The author declares that the research was conducted in the absence of any commercial or financial relationships that could be construed as a potential conflict of interest.
